# Saving explorers and healing Amazonians: a history of the Tabloid medicine chest used in Hamilton Rice’s 1919-1920 Amazon expedition

**DOI:** 10.1590/S0104-59702025000100047

**Published:** 2025-10-20

**Authors:** Victor Rafael Limeira-daSilva

**Affiliations:** i Researcher, Museu de Astronomia e Ciências Afins. Rio de Janeiro – RJ – Brazil. victorrafael@mast.br

**Keywords:** Pharmacology, Tabloid medicine chest, Capitalism, Exploration, Amazon, Farmacologia, Caixa de remédios Tabloid, Capitalismo, Exploração, Amazônia

## Abstract

This paper draws connections and parallels between the promoted objective of Burroughs Wellcome & Co.’s Tabloid medicine chests and the capitalist and scientific ideals related to modernising the Amazon, which were advanced by the physician and geographer Alexander Hamilton Rice. Using an object-biographical approach, I explore the context of the promotional discourse surrounding the Tabloid chests and detail the contents of the medicine chest Rice carried throughout his 1919-1920 expedition, considering the pathological imaging fostered by the company and its customer. The relationships between this marketing discourse and Rice’s search for scientific recognition and publicity as a quintessential explorer are also analysed.

## Resumo

Este artigo estabelece conexões e paralelos entre o objetivo promovido pelas caixas de remédios Tabloid da Burroughs Wellcome & Co. e os ideais capitalistas e científicos relacionados à modernização da Amazônia, defendidos pelo médico e geógrafo Alexander Hamilton Rice. Utilizando uma abordagem biográfica de objeto, exploro o contexto do discurso promocional em torno das caixas Tabloid e detalho o conteúdo da caixa de remédios que Rice carregou durante sua expedição de 1919-1920, considerando a imagem patológica promovida pela empresa e seu cliente. As relações entre esse discurso de marketing e a busca de Rice por reconhecimento científico e publicidade como explorador por excelência também são analisadas.

The British pharmaceutical giant Burroughs Wellcome & Company (BWC) has been extensively discussed in general terms in historiography. One of the most important aspects writers address is the company’s role in fostering British neo-colonialism through the new global commodity culture it helped shape. As for its medicine chest product line, researchers have also shown that the manufacturing and advertisement of medical kits for explorers and missionary doctors reinforced earlier British ideas of “natural superiority” over people in colonised territories. Historians have called attention to the role of the company’s grounding scientific knowledge and commercial model in guiding twentieth-century British views of the “tropical world,” its people, and its social health conditions, while the successful trajectory of BWC’s pharmaceutical laboratories led to discussions on the role of trust in the development of science and pharmacology in the early 1900s (Hill, 2006; Church, 2006; Johnson, 2008a, 2008b; Beckett, 2008; Larson, 2009, p, p.63-127).

Important works have also been written on the history of medicine chests, as historians have been exploring domestic healthcare practices and instruments for some time. This scholarship includes the earliest European medicine chests on record, from the sixteenth century, most notably the origin and broader context of the Giustiniani medicine chest at the Science Museum London Collection, where it arrived through the Wellcome Collection after being acquired in Italy in the 1920s (Ackroyd, 2019). Eighteenth- and nineteenth-century medicine chests (the predecessors of modern bathroom medicine cabinets) have also appeared in historiography, with studies investigating the evolution of treatment and cure methods and the history of “pre-scientific” medical instruments and practices such as enemas, piercing, bloodletting, and purging (Crellin, 1979; Young, 1994).

More contemporary medicine chests (like the Tabloid) have also been addressed in several publications. Some studies focused on understanding the history of material culture have pointed out how BWC’s medicine chests embodied notions of advancement and modernity. By fomenting state-of-the-art pharmacology and exhibiting these objects (as the remnants of triumphant or disastrous explorations), the company reinforced an underlining evolutionist narrative of the history of science and medicine (Hill, 2006; Craciun, 2016). Meanwhile, other historians have argued that the BWC medicine chests were not as pioneering as the company claimed them to be: they featured drugs that had been in use for centuries in various contexts, revealing that the “civilisational mission” of these chests often depended on medicines already in use in the very places Europeans were supposedly civilising (Johnson, 2008b). Undoubtedly, even analyses that question BWC’s discourse on the pioneering spirit acknowledge the mobility of the medicine chests as an effective novelty for the development of a new commodity culture that combined science, healthcare, industry and commerce.

At the same time, the history of the medicine chest used by the American doctor and geographer Alexander Hamilton Rice Jr. (1875-1956) in one of his famous medical/geographical expeditions to the Amazon (1907-1925) has not yet been analysed in detail. In this paper, I draw connections and parallels between three dimensions of this historical case: (a) the promoted mission of the BWC Tabloid medicine chests; (b) the ideals of capitalist and scientific modernisation and improved health of Amazonian society promoted by Rice, one of BWC’s most famous customers; and (c) Rice’s vocational rhetoric and search for scientific recognition and publicity. First, I contextualise BWC’s business, paying special attention to examples of the discursive dimension underlying the process of promotion for their line of medicine chests, notably by supplying them to celebrities of science and exploration. Next, I focus on the Tabloid medicine chest itself, detailing the contents of this item Rice carried to the Amazon in the 1910s, with two objectives: to understand the pathological scenario in the Amazon (fostered by both BWC and the explorer) and to comprehend how the chest’s dual profile as a medical/scientific and commercial object fit with early twentieth-century notions of science relics. Finally, turning to Rice’s fifth Amazonian expedition, I analyse the relationship between the promotional discourse for the Tabloid medicine chests and the ideas underscoring Rice’s self-proclaimed mission of scientific modernisation and Amazonian exploration as well as personal recognition.

This study offers fresh contributions to two key themes in the historical study of science and exploration. First, it addresses the historiography of medicine chests by examining this case of an important scientific fieldworker and customer of medicine chests whose exploratory profile, scientific ambitions, and values embodied BWC’s “civilisational” aspirations. This intricate case also sheds new light on the historiography of medicine chests in a novel context, between the North American, British, and South American transnational scenarios of scientific and commercial circulation, since most of the specialised literature thus far has focused on the Arctic, Antarctic, African and Asian contexts. This study is also novel for bringing together the histories of the science explorer and pharmacological company through analysis of the discursive and effective capacity of mobility, as well as the scientific and technical reliability of a medicine chest. Second, this research contributes to the historiography of Rice’s Amazonian trajectory in geography, medicine, and philanthropy, considering its entanglements with capitalist ideals of modernisation through scientific and medical field research. This article deepens our understanding of Rice’s endeavours and their relation to the history of BWC’s medicine chests while it considers Rice’s vocational strategies and effective scientific and medical efforts. The research that forms the foundation of this study also enriches Rice’s scientific biography, since it explores his ideas and activities from a specific and meaningful object that was part of his array of new technologies for travel, healthcare, and geographical exploration which was central to his self-assertion.

## Burroughs and Wellcome’s Tabloid medicine chests

Henry Solomon Wellcome (1853-1936), one owner of Burroughs Wellcome & Company, researched, collected, and designed many varieties of portable medical kits found around the world known as “medicine chests.”^1^ After graduating from the Chicago and Philadelphia Colleges of Pharmacy in 1874, Wellcome moved to London, where in 1880 he co-founded the company with another American, the entrepreneurial pharmacist Silas Burroughs (1846-1895). Together they pioneered compressed drug tablets in Britain, created a great range of other pharmaceuticals and chemicals in general, and took marketing ideas to a whole new level of shrewdness. As a result, by 1914 BWC had become Britain’s leading drug company (Church, 2006, p, p.376).

To improve and promote its medicine chests, the company was particularly interested in tropical territories, despite the notoriety of Arctic and Antarctic expeditions in advertisements and exposition guidebooks (BWC, 1934, p.11, 62-63; Guly, 2015). The tropical regions were considered a particularly threatening environment for European explorers, as the company made clear in its hyperbolic promotional materials:

A danger far worse than that of broken limbs, of cuts and gun-shot wounds, hangs over the traveller in remote places, particularly in the Tropics. The worst menace he has to face is disease. In those regions a mere scratch on a finger can produce an immediate infection ... The Tropics … are by far the most dangerous regions for travellers. There, in addition to the ever-dangerous scurvy they encounter such desolating ailments as black fever, yellow fever, dysentery, typhoid, sleeping-sickness, beri-beri and the ever-present smallpox. All of these are particularly fatal to the so-called white man … In addition, there is malaria, which, although it occurs in temperate climates, is much more prevalent and virulent in the Tropics. In some of these far-off places, the natives have a degree of immunity to certain diseases. But that immunity is not enjoyed by invaders from the temperate zones. The ravages from these diseases up to some 50 years ago, as we shall presently see, were terrific (BWC, 1934, p.7-9).

Special attention was paid to the tropics in advertisements and guidebooks, since the very existence of diseases was sufficient marketing justification for the company and these regions featured many British colonies and commercial outposts. Furthermore, exploration of these areas justified selling the chests while simultaneously providing rich sources for appropriating local natural medicines. BWC claimed to be grounded on a global conception of market and state-of-the-art scientific research and also inspired by government calls for British companies to always “work imperially” (BWC, 1912, p.29; Johnson, 2008a, p.71).

Around 1883, Wellcome began to research medicine chests and cases from different regions and eras in detail. His objective was to establish the company’s own line of these products, taking advantage of the remaining British colonial territories and the number of foreign expeditions that continued to proliferate. But it was initially Burroughs’s idea to include medicine chests in the catalogue of Tabloid products, reinforcing the point made by Julia Sheppard (2022) that he was the real “brain and energy” behind BWC’s success before his early death. In any case, as Wellcome pursued raw materials and techniques to improve the company’s chests, he also planned to include their own products amongst the historical medical artefacts BWC exhibited at fairs, conferences and expositions (Larson, 2009, p.16-19). This was an astute marketing ploy, since the stands were visual and material statements on the already groundbreaking nature and historic status of the company’s chests (Beckett, 2008, p.111).

The name Tabloid (referring to the compressed structure of the medicine capsules) was applied to a range of other BWC pharmaceutical products and photographic chemicals. By the 1910s, the Tabloid brand had already taken the lead in the company’s sales and massive advertisements. The Tabloid chests, which contained all the drugs and surgical material required for any kind of journey by tourists or professionals, were mainly expected to modernise the experience of explorers, missionary physicians and travellers in general in distant parts of the world. With its diversified set of partners around the globe, BWC usually made the pretentious claim that the company was also elevating the general health standard in “unhealthy” locations (BWC, 1912, p.41, 1924, p.1-2; Johnson, 2008a, p.70). This discourse also interested the company’s client, the medical explorer Hamilton Rice, as we shall see further on.

The chests were considered essential for saving Europeans from “foreign” diseases, but also understood as instrumental in healing Indigenous collaborators and other locals, given their mobility and practicality. As Ryan Johnson (2008b, p.258) recalled based on David Arnold’s geographical conceptual notion of “tropicality” (Arnold, 2000), BWC’s medicine chests helped cement the boundaries between European “civilised identity” and its polar opposite, represented by the “primaeval” and “unhealthy” tropics and their people. Nancy Stepan’s conceptualisation of the term “tropical” also offers even deeper insight into the role of BWC’s medicine chests as part of this differentiation process that grounds European colonial thought (Stepan, 2001, p.13). Expanding the discursive representation of the tropics to a variety of “images of tropicality” makes it possible to imagine many other cultural and social areas in which Europeans and North Americans shaped the tropical panorama, such as the health and social imagery of the tropics spread by Wellcome’s industrial pharmacology and cemented by Hamilton Rice’s salvage medical-geographical discourse, as we shall see.

Even before the establishment of his Historical Medical Museum in London in the 1910s, Wellcome’s antiquarian, scientific, collecting and capitalist interests were intertwined. In addition to investigating the history of medical practices in various cultures, he oversaw archaeological excavations, travelled to collect historical and ethnographic objects, and transformed the company’s fair exhibits into historical and ethnographic showcases of healthcare (Hill, 2006, p.366-367). The company was keenly aware of how it could capitalise on the growing exhibition culture of commercial displays. In the wake of major international fairs and world exhibitions in the 1800s, the medical product displays created by BWC redefined the meanings associated with the scientific and commercial roles of its medicine chests within the context of exploration cultures. While the product and technology exhibits at events like the Great Exhibition of 1851 in the Crystal Palace reflected general national pride and progress, BWC’s exhibitions and the Wellcome Collection placed greater emphasis on the brand and the “genius” behind their groundbreaking products.

The company’s main marketing strategy consisted of supplying the chests at no charge to renowned or promising explorers, publicising their reviews of the products, and displaying the chests (returned after the expeditions) as historical artefacts in major exhibitions (Larson, 2009, p.30-31). As Adriana Craciun (2016, p.35) explained, the practice of procuring, collecting, and advertising objects from celebrated expeditions as “science relics” dates back to the mid-century Victorian culture around relics of disasters, such as Franklin’s lost Arctic expedition (1845-1846). More importantly, most of those objects (whether medical, scientific, or technical) were understood as “traces” or “relics” of science and exploration even before they had been publicly displayed; this was partly due to the fact that this culture shared common ground with antiquarianism and archaeology, which became common bourgeois hobbies from the mid-nineteenth century. It is well-known that the BWC was very interested in these two collecting traditions. For example, by classifying the returned chests as “relics” in the Souvenir of the Colonial British Empire Exhibition of 1924, the company emphasised that “the Medical Equipments of all the great Expeditions are supplied by Burroughs Wellcome & Co.,” a mere truism or a “typical instance” (BWC, 1924, p.8). Collecting the company’s own chests and displaying them as relics of modern science and exploration became BWC’s *modus operandi* for advertising its most successful products. This is how the medicine chest used by Hamilton Rice in the Amazon ended up in the Wellcome Collection around 1920 before being transferred to the Science Museum in the 1970s, as we shall see below.

Some medicine chests in the Tabloid line were branded after the most famous explorers; the Livingstone sub-line was launched in the 1910s, taking advantage of the exemplarily “heroic” history of the Scottish colonialist, physician and missionary David Livingstone (1813-1873), who was thought to have gone missing during his second expedition to Central Africa in the 1860s. Although the journalist and explorer Henry Morton Stanley (1841-1904), who led the expedition to find Livingstone in 1871, was not a BWC client until his final expedition in the 1880s, he became one of the best known “poster boys” and promoters of the firm. Frequently quoting Stanley’s firsthand analysis that the “crude way in which medicines were supplied to travellers” was the main reason for their high mortality abroad, BWC proudly stated that “it was left to Burroughs Wellcome & Co. to remedy this state of things” (BWC, 1901, p.2, 1928, p.4-5, 1934, p.23-4; Johnson, 2008b, p.255).

Another interesting instance is related to the Emin Pasha medicine chest, also from the Tabloid line. Originally supplied by BWC to the Ottoman physician and governor of the Egyptian province of Equatoria for whom it was later named, the chest was stolen twice before being recovered in 1898 near Kenya and returned to BWC in London (BWC, 1928, p.5-6). The history of this chest further exemplifies the “personality” of the product that resulted from BWC’s clever marketing strategy (Johnson, 2008b, p.255).^2^ When news of the chest’s return was made public, the British media published relatively broad coverage of its African peregrination. Newspapers were flooded with headlines like “The adventures of a medicine chest,” attributing the object its own history (The adventures…, 12 Nov. 1898; Emin…, 17 Nov. 1898). Although medicine chests were primarily intended to store medicines to preserve their efficacy, portability was the most notable feature in both marketing and public perception. The idea of modern, dynamic health treatment made possible through science-based pharmacology and medicine was central to the success of medicine chests like the Emin Pasha. This concept of universality, embodied in the material form of the medicine chest, played a key role in media coverage and its commercial appeal.

Tabloid medicine chests also received nicknames referring to regions where their use was associated with recent explorations. For instance, the Congo medicine chest – the same line and model as those used by Stanley and Hamilton Rice – was a nod to the chest used by American diplomat and explorer Richard Dorsey Loraine Mohun (1864-1915) in his expeditions in the African Congo between the 1880s and 1910s. Another example is the Indian chest, advertised as “specially adapted for Indian and Tropical use.” This was a reference to the journalistic travels of George Warrington Steevens (1869-1900), who became the most famous British war correspondent of that time. He used Tabloid medicine chests during the war campaigns in Greece, Sudan and South Africa and during his trips throughout India (BWC, 1928, p.22-23).

In a letter to Wellcome, Burroughs (1882, p.5) stated that the medicine chests and cases were not only products but also the company’s best marketing strategy, since they would sell and advertise themselves when “in the hands of each doctor and chemist,” spreading “our goods over the world in a hurry greatly to our credit and profit.” These examples highlight that this strategy was adopted by BWC, which heavily invested in the “personalisation” and movable character of the medicine chests for its advertising. The marketing discourse granted the chests the biographical status of witnesses to the most important exploratory, medical, and scientific achievements of the era, which fostered their potential to be seen as “relics” of science and exploration (BWC, 1934, p.68).

The history of the BWC medicine chests points to the relationship between product sales, advertising, and the cultures of scientific exploration in the early twentieth century. Stemming from an earlier model of commodity understood as an idealist, placeless form that shapes exchanges, capitalism developed a new commodity culture between the late nineteenth and early twentieth centuries. According to Thomas Richards (1991, p.1-2), “the fundamental imperatives of the capitalist system became tangled up with certain kinds of cultural forms, which after a time became indistinguishable from economic forms.” The most characteristic dimension of this renewed commodity culture is the fact that it became dominated by advertising, which was consequently fed by the culture of spectacle and exhibition that marked the ethos of imperialist societies like Britain. Given that the already established tradition of scientific exploration was also a fundamental part of this milieu, we can further understand that the history of creating, advertising, collecting, and exhibiting the BWC medicine chests exemplifies how pharmacological science and industry embodied the meanings of colonialism, civilisation, advancement, and modernity through innovative cultural forms such as objects and instruments with overlapping economic and scientific meanings.

## Rice’s Tabloid medicine chest

Sir Henry Wellcome’s Museum Collection of historical medical objects held by the Science Museum London contains a set of medicine chests carried by scientists, explorers, politicians, military and missionaries on famous journeys in different parts of the world. One such chest, a Tabloid from the Congo sub-line, was used by the American explorer Hamilton Rice in his 1919-1920 expedition to the Negro and Orinoco rivers and their tributaries in the northwestern Amazon. Its standard market price was around £24, the equivalent of approximately £1,500 in today’s currency, hardly an affordable item (BWC, 1901, p.35).

The case itself, its wings and lock are made of aluminium, while the inner case is made of wood. Its lid is divided into two sections, designed with a space to store surgical and first-aid outfits, bandages, cotton, syringes, and other medical accessories. The case itself was originally filled with forty bottles made of gutta-percha,^3^Bakelite, or glass containing various Tabloid, Soloid, and Warburg brand drugs, other BWC pharmaceuticals, and a Medical Guidebook (BWC, 1901, p.35). It weighs a total of 9.86 kilograms and is no larger than a medium-sized parcel box.^4^ As for the specimen’s current state of preservation, the right-wing hinge is broken and the right section of the lid does not open. For safety reasons, the controlled drugs have been removed from the chest and placed in restricted storage by the Science Museum (Science Museum Group, 2012).

**Figure 2:**
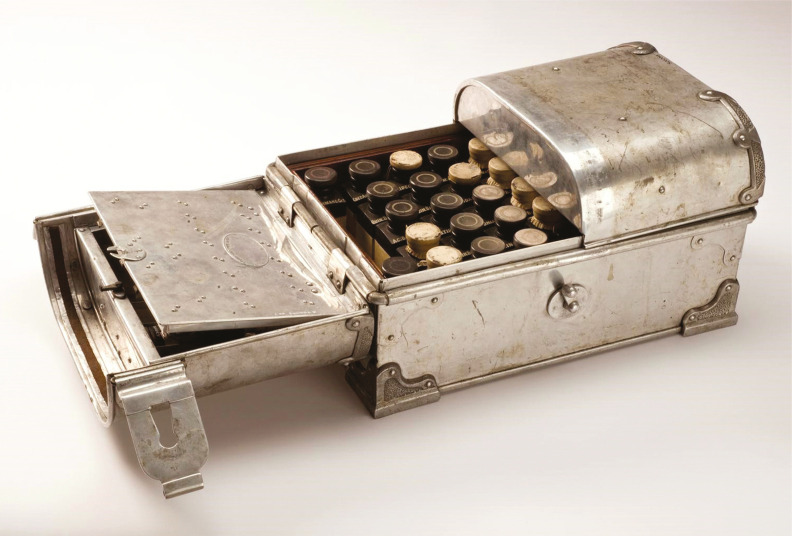
: Tabloid medicine chest used during Hamilton Rice’s Amazonian expedition in 1919 (© Science Museum Group Collection. CC BY-NC-SA 4.0)

The chest contained the following medications: bottles of chloroform and morphine tincture, for sedation; bottles of ipecacuanha and squill tablets, used as expectorant; tubes of asafoetida and opium compound, mainly for use as a digestive aid; canisters and bottles of opium extract, used for millennia as a sedative and analgesic; bottles of camphorated tincture of opium (paregoric), mostly used at that time to treat diarrhoea; bottles of Warburg brand tincture, a famous antipyretic used for treating tropical fevers; bottles of aromatic chalk powder with opium, usually used on wounds as a painkiller; tubes of kino and opium compound powder for skin-related infections; quinine and iron-based syrup and tablets, widely used for treating malaria; aspirin tablets, at that time known only for their analgesic properties; and sleeping pills, most likely chloral hydrate-based (synthesised for sedative use in the second half of the nineteenth century) or morphine-based (Science Museum Group, 2012).

Most immediately notable about the contents of the chest is the great quantity of opium-based medicines, which is quite understandable considering that the history of opium and opioids, as addictive and widely disseminated drugs, is intertwined with human history itself. But other than the drugs used for common respiratory, digestive, sleep, and skin conditions, the majority of the medications are quinine-based drugs and solutions. Clearly, malaria was one of the most common tropical diseases and was still treated mainly with quinine at that time, and considered a great danger for people from temperate zones who ventured into tropical regions. According to Sandra Caponi (2006, p.277), as modern tropical medicine emerged in the early twentieth century, malaria was the “protagonist” of the pathological scene. The high mortality rate associated with malaria and its epidemiological pattern turned it into the “paradigm of tropical disease.” But from the early twentieth century, first Brazilian and then German doctors and researchers concluded that many cases were quinine-resistant, paving the way for experimental treatments with other drugs like atoxyl. Still, even as late as the mid-1940s, some doctors maintained that no single drug could completely cure malaria (Silva, Benchimol, 2014, p.2; Benchimol, 2018, p.12-17).

The presence of Warburg tincture in the chest is also interesting, since from the late nineteenth century this treatment was considered superior to pure quinine-based drugs for treating tropical fevers, although it also contained quinine in its composition (Maclean, 13 Nov. 1875, p.718). In any case, the quantity of quinine-based drugs in Rice’s medicine chest is also explained by the fact that this alkaloid, extracted from the bark of the South American cinchona tree (*Cinchona pubescens*), was also used preventively. Overall, most of the drugs in the chest were intended to treat muscle pain, fever, nausea, headaches, and diarrhoea, all symptoms of the most common tropical diseases. The remainder were clearly intended for medical or surgical needs related to the exploratory or travel experiences themselves, such as wounds, cuts, broken limbs, and skin infections.

**Figure 1:**
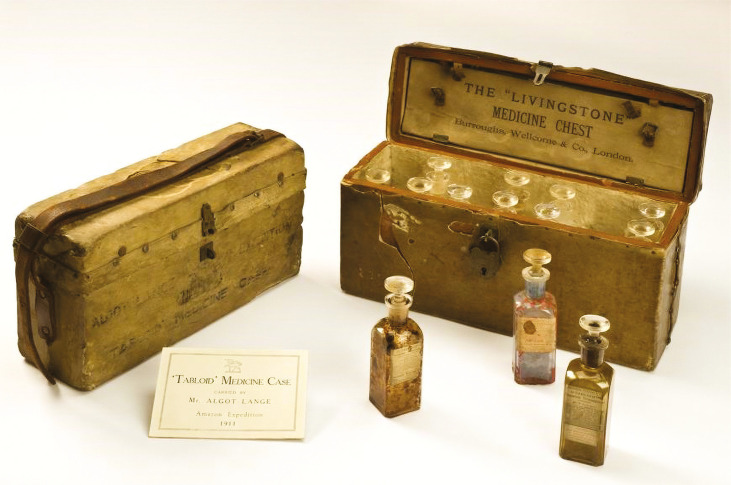
: The Livingstone Medicine Chest used by Algot Lange on the Amazon expedition of 1911 (© Science Museum Group Collection. CC BY-NC-SA 4.0)

From the perspective of “object biographies,” each feature, mark, value, person, or meaning attributed to the history of an object plays a potential role in better understanding the surrounding events in which it took part or the object itself (Alberti, 2005; Hill, 2012). The case of Rice’s medicine chest is no different, especially because of its contents, which reinforce a view that questions some of the marketing discourse related to BWC’s pioneering medical achievement. Not only were most of the abovementioned medicines not a novelty for everyday people in South America or anywhere else, but they had also been a part of the experience of travelling to the tropics for centuries. Moreover, the chest and the drugs it contained show that both BWC and Hamilton Rice held a pathological view of the Amazon region that was essentially the same classical medical image of “tropicality” promoted by Europeans for centuries. Nevertheless, what can be argued in favour of BWC’s propaganda is that the proportion of each drug, the compactness of the chest and medicine bottles, the material from which the case was made, as well as the mobility of the chest clearly show that it was well-suited for explorations in the Amazon. On this point, it is important to mention that an aluminium case is much more resistant to rust and mould in the humid forest environment than one made of other materials, and better preserves its contents. This is clear when comparing the conditions of Rice’s aluminium chest to the wooden one carried by the Swedish-American explorer Algot Lange (1884-1961) to the same region a few years earlier (see Figure 1).

Although we cannot provide many details about the route Rice’s medicine chest travelled back to BWC or its entry into the Wellcome Collection, some information is available. In the “Points for Propaganda” in the BWC memoranda for the years 1919-1921 some mentions are made of the marketing and exhibition potential of Rice’s medicine chest. These memoranda were primarily meant for sales staff, providing updates about products and sales campaigns. The files classify the chest in the “Brazilian explorers” section, including its features, Rice’s report on the use of the product, and some notes on the other medical equipment he carried on his fifth Amazonian expedition (Monthly…, 1919-1921).

It is consequently quite certain that Rice’s medicine chest was returned to BWC as soon as the expedition ended in April 1920, and entered the Wellcome Collection soon after. Mentions of Rice’s chest and expedition in the BWC papers show that Wellcome considered the object to be a potential inclusion in the company’s relics since it was supplied to the explorer. In the 1910s, Rice already commanded a reputation amongst American and British geographers, physicians and other scholars, although this reputation was problematic for some of his geographical peers, as we shall see in the next section. In any case, Wellcome had known Rice since at least 1913, when he provided complimentary tickets to the Historical Medical Museum. In an enclosed letter, Wellcome demonstrates that Rice was very interested in medical historical displays, which also helps explain the presence of medical equipment from Rice’s later travels in the Wellcome Collection (Wellcome, 14 Oct. 1913). A long-term loan brought thousands of objects from the Wellcome Collection to the Science Museum London since the 1930s, including Rice’s Tabloid medicine chest, which has been kept there since 1977. More than three thousand objects constitute the permanent exhibition “Medicine: The Wellcome Galleries,” which opened in 2019 but unfortunately is not displaying Rice’s medicine chest.

Having described and understood what the Tabloid medicine chest reveals about the views, interests, and expectations of its creator and user, it is now important to conclude this section by pointing out why this particular medicine chest became a relic within the context of cultures of exploration, commodity and exhibition. According to Craciun (2016, p.36):

The closest analogues to these [science and exploration] relic displays were the anthropological exhibitions of other cultures, and the commercial displays and advertisements coeval with the rise of department stores, trade exhibitions, and arcades. Neither of these exhibitionary cultures – the anthropological or the commercial – had desirable associations … . They would emphasize the relics’ affinities with the sacred and the scientific, but the relics would stubbornly maintain their unwelcome associations with commodities and exotic artifacts.

Interestingly, the author’s main analytical context reveals that in the case of relics from disasters of science and exploration, the overlapping ethnographic and commercial meanings were generally avoided. This was not the case for the background of Rice’s Tabloid medicine chest, however: this medical kit played a key role in new, ingenious forms of collecting and displaying products from an industry that simultaneously relied on science and fuelled scientific research. The commercial value of the object came to constitute the very reason why it returned home and was certified by Wellcome as representative of scientific modernity and capitalist creativity. Moreover, the museum’s biography of the Tabloid medicine chest still emphasises the fact that BWC medicine chests were amongst Rice’s discerning top choices for modern travel and exploratory technologies (Science Museum Group, 2012), which constituted the crux of his whole vocational rhetoric, discourse of authority, conquered recognition and fame, as we shall see.

The context analysed by Craciun (2016) was shaped by Britain’s response to the economic impacts of the First World War and the Great Depression, which followed a period of visible economic fragility. The exhibition and marketing cultures in which Rice’s medicine chest was presented and promoted were part of a broader movement aimed at capitalising on new colonial possessions secured through wartime treaties, all in an effort to boost declining exports. Investing in medical products that could support the business of exploration during this era of a rapidly changing British Empire was a very welcome move. There is little doubt about why these medicine chests belonging to Rice and other notable figures quickly became regarded as scientific relics while also holding significant commercial value.

## Rice’s fifth Amazonian expedition

The history of BWC’s Tabloid medicine chests draws interesting connections with Hamilton Rice’s trajectory of self-construction as a celebrated explorer of the Amazon. Rice received a medical degree from Harvard Medical School in 1904 before serving in France during the First World War and as a surgical officer at the Massachusetts General Hospital. As a multi-millionaire, he was able to relinquish other professional attachments and dedicate himself to various medical-geographical journeys (first in Canada, followed by Eastern Europe, Russia and South America) from his twenties to his fifties. Rice completed seven expeditions in the Amazon region in Ecuador, Venezuela and northwestern Brazil between 1901 and 1925 (Martins, 2012, p.225-226), which became the stepping stones to his later scientific recognition.

After his first Amazonian journey, he spent three years studying for a diploma in professional cartography from the Royal Geographical Society (RGS) in London (1910), under the instruction of Edward Ayearst Reeves (1862-1945). Rice’s personal goal was to improve his knowledge and surveying practices as much as possible. As authentic as these scientific interests might have been, this pursuit of RGS support was also an attempt to dodge some questioning of his effective scientific capacity and credentials. Some scholarly circles considered Rice more of a publicist than a real scientist, criticisms he was unable to fully overcome even at the height of his career (Martins, 2012, p.225).

This did not, however, mean that many other scholars and institutions were not convinced by Rice’s scientific authority. He founded the Harvard Institute of Geographical Exploration, and was appointed its first professor in 1929. Yet his appointment was questioned by some peers who believed that a huge donation from his wife to the institute’s foundation was the real reason behind his academic ascension. Although some scholars did not find him particularly convincing, Rice was very well-connected. He was married to the socialite Eleanor Elkins Widener (1861-1937), a wealthy survivor of the Titanic shipwreck. Rice himself was heir to the fortune of his grandfather and namesake, a businessman and politician who served as the mayor of Boston, governor of Massachusetts, and US congressman. Unfortunately for Rice, the Harvard Institute closed in 1952, after its results were considered undefining for the modern development of geographical teaching and research (Martins, 2012, p.236, 239).

Rice nonetheless worked to make his series of expeditions examples of the scientific modernisation of the Amazon, the ways of exploring it, and health treatment. His goals were very similar to BWC’s views regarding the role of their products in fostering the mobility of medical treatment during colonial, scientific and capitalist enterprises. It is also important to note that even in terms of publicity strategies, the explorer/customer and the company shared common ground. Whereas BWC made the most of the pioneering mobility and reliability of their chests in their marketing campaigns, Rice spent a lot of time building the idea of an unmatched career and reputation in geography and medical research, in large part also fostered by publicity. Counting on high-level supporters in the RGS, Rice was honoured with the Patron’s Gold Medal in 1914, although the justification for the award was quite vague and lacked effective academic motivations: “For his meritorious work on the headwaters of the Orinoco and the Northern tributaries of the Amazon” (RGS, 1914, p.107). He also was able to join the RGS as a Corresponding Fellow. In the United States, he was awarded the David Livingstone Centenary Medal by the American Geographical Society upon the completion of his fifth expedition to the Amazon in 1920.

Unsurprisingly, Rice became quite obsessed with equipping his expeditions with the most advanced gadgets to explore, map and study the Amazon in a way he considered far superior to his nineteenth-century predecessors (Rice, 1920, p.59-60; Grann, 2009, p.91; Martins, 2012, p.225-226). His 1917 expedition included a modern motor-powered launch to overcome the most arduous stretches of river, while his 1919-1920 expedition pioneered wireless radio communication in the upper Negro river, receiving updated coordinates and maintaining near-daily contact with counterparts and family in the United States. Rice was also the first to survey and take aerial photographs of the Amazon region with a floatplane, remapping areas presented in previous cartography. His choice to include the Tabloid chest amongst the equipment for his fifth expedition was consequently due to his already-vast knowledge of the region, notably the most prevalent diseases in the Amazon, which he knew could prevent his team from further advancing inland. Moreover, considering Rice’s taste for publicity (as we shall see in detail), associating his expedition with the BWC propaganda certainly must have sounded interesting.

**Figure 3:**
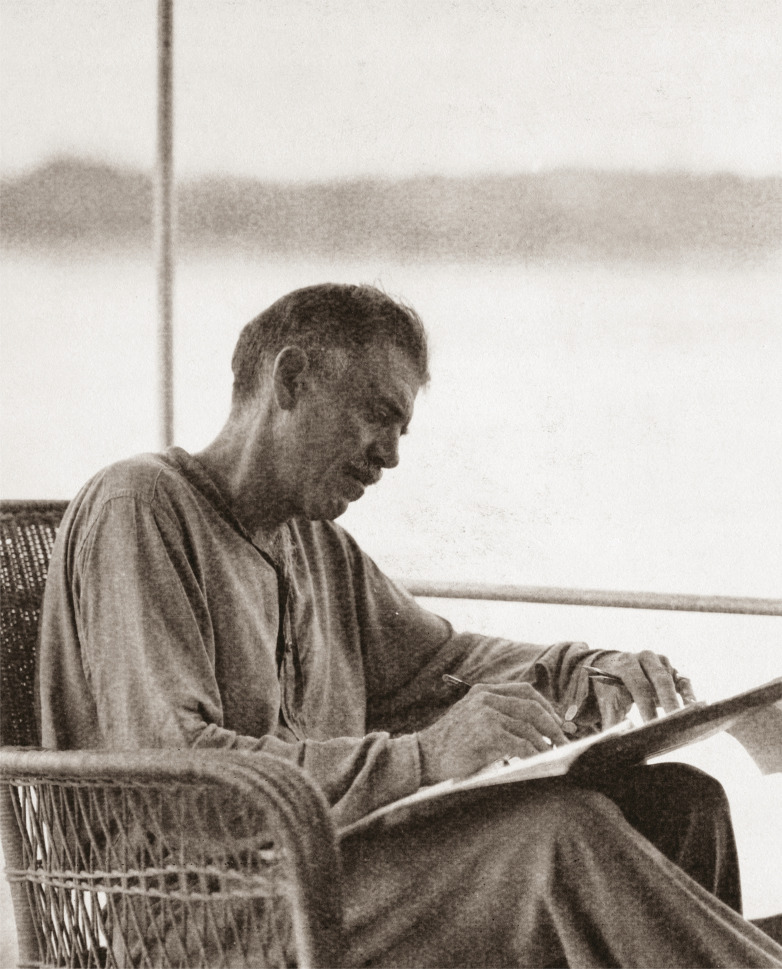
: Alexander Hamilton Rice, Brazil, circa 1919 (Hamilton Rice Expedition 1919-1920; by permission of the © Royal Geographical Society)

Even though Rice made himself known for geographical surveying, the central goals of his series of expeditions were also medical, philanthropic, and ethnographic. Rice believed that the real problem in Amazonian society was the lack of healthcare and education. He thought he could pave the way for high-tech explorations of the Amazon while planting the seeds of a healthier and civilised society. Although the people in Amazonia showed great potential for becoming “civilised,” Rice maintained there was still a long way to go. In his opinion, the first steps to be taken in his expeditions were to investigate endemic diseases and introduce medical assistance with cutting-edge drugs and grounding scientific knowledge (Councilman, Lambert, 1918).

Although Rice did not appear frequently in BWC’s advertisements, his journeys (which were enthusiastically covered in British and American scientific journals and newspapers) were the type of enterprise the company expected its Tabloid chests to be associated with. In addition to Rice’s fame, his reports on the pathological scenario in the Amazon and Indigenous medicines contained the kind of data the BWC research laboratories strongly desired. As mentioned in the first section of this article, although large-scale production of the medicine chests was standardised, BWC specifically invested in one of their most important features: the ability to personalise a chest to bolster its capacity to move around the globe. For the company’s marketing, it was very important to show that the destination of the client was no impediment, given the wide range of drugs and accessories contained in their chests which were suited to each climate and health condition in the world (BWC, 1901, p.9).

As a medical customer, it is likely that Rice himself required a specific type of chest from BWC, with distinct drug dosages and surgical accessories he needed. Indeed, as noted earlier, since his 1914 expedition Rice had dedicated significant effort toward studying the diseases of the northwestern Amazon and describing the sanitary conditions amongst the people living there (Rice, 1914, p.140). Although most of the pathological research was conducted by his medical team, composed of the physicians William Thomas Councilman (1854-1933) and Robert Archibald Lambert (1883-1960), Rice analysed 250 cases of malaria and presented the mortality rates for numerous villages and towns, paying special attention to the medical entomology in the areas with higher figures (Rice, 1914, p.142). Though the transmission of malaria had already been discovered in 1899, details regarding the protozoan and treatments were still under investigation (Benchimol, Silva, 2008, p.720-721; Benchimol, 2018, p.2). Rice took various notes about the possible relations between the local diet and prevailing diseases, and took advantage of killed animals to track the prevalence of the agent of malaria, the *Plasmodium* parasite (Rice, 1914, p.142-143).

Like Wellcome, Rice focused on two main targets: his exploratory team, which included over 100 men and his wife in 1919, and the people of the Amazon. A classic entrepreneur and philanthropist of the early twentieth century, his main preoccupation was the future of capitalist civilisation and scientific advancement, which he believed had been delayed for local historical and political reasons, but also and especially because of what was considered the hostile environment, which killed many more foreigners than Indigenous people and other locals. Both Rice’s writings and BWC adverts often referred to earlier explorers, arguing that precarious healthcare and instruments available to celebrated figures like Alexander von Humboldt (1769-1859), Johann Baptist von Spix (1781-1826) and Karl von Martius (1794-1868) in South America and David Livingstone in Africa prevented them from achieving some of their main objectives, and almost drove them to death (Councilman, Lambert, 1918, p.103-104).

During Rice’s fifth expedition, the pages of *The Geographical Journal* (a periodical published by the RGS) were filled with firsthand accounts of his achievements. Although the geographical feats were most obvious, Rice’s fame also stemmed from his work with tropical diseases and the “limited” social and technological situation in northern Brazil. Interestingly, the discursive bottom line of Rice’s series of expeditions was precisely the modernisation of the geographical surveying experience, which obviously involved upgrading the logistics of transporting, maintaining, and administering medicines and medical assistance in general throughout the journey. For Rice, the BWC medicine chests and other top-notch survey gadgetry materialised this discourse. It is curious and ironic that despite major achievements in exploration and being surrounded by high-tech paraphernalia, Rice’s general exploratory style and scientific approach were considered by some geographical peers to be quite old-fashioned for geography. According to Luciana Martins (2012, p.239), “Rice’s ideal of what constituted geographical science was … grounded in nineteenth-century models of geographical exploration.” Nevertheless, some resistance to Rice’s methods and technology is completely understandable, since geoscience in general was a very well-established scientific field since at least the late eighteenth century. Attaining status in such a standard scientific arena was therefore no easy task, even for someone with the means to do so. For some, it seemed as if Rice stubbornly wanted to earn the same kind of recognition granted to contributions on the level of Humboldt, Livingstone or Alfred Wallace (1823-1913), while enjoying an easier and more comfortable surveying experience. The same is true for comparative understanding of BWC’s constant search for public recognition of its medicine chests. Standing on the shoulders of many others who brought about most of the medicines in these chests, the company heavily promoted the revolutionary transformation these medicine chests and cases afforded in terms of mobility and practicality in administering these medications.

Although Rice faced some stumbling blocks along his road to recognition in geography, his credentials were very much respected in medicine. The available examples show that it was his use of BWC products that helped solidify his medical reputation while in the field. Reports of his 1919 expedition enthusiastically stated that he performed the first surgery using the anaesthetic chloroform in the Amazon when he operated on Amali, a Huitoto woman and the wife of Cristobal (his guide in Colombia) who was suffering from a liver abscess.^5^ He also reported treating a man from the banks of the Ariari River whose kidney had been perforated by the lance of a Yamu man.^6^ Rice was also celebrated as an industrious surgeon for operating on his own leg to treat phagedenic ulcers, which were believed to be caused by many agents but in his own opinion were undoubtedly fostered by the climate and sanitary conditions (Rice, 1914, p.149-150). Comments on his report of the 1919 expedition also praised him for “the tact he showed in getting his men together and contented, and the remarkable way in which he looked after them in preserving them from every kind of fever” (Gama, 1921, p.344). In 1919, Rice also provided medical and surgical treatment to his sorely ill host, Antonio Castanheiro Fontes, a landowner living in Umarituba, Amazonas, along with his household (Rice, 1920, p.60). All these feats were achieved using BWC surgical accessories and drugs, which the company would soon make the most of as part of its marketing strategy.

BWC played its part in publicising the merits of its explorer/customer. In its 1930s book project entitled *The Romance of Exploration and Emergency First-aid from Stanley to Byrd*, Rice was credited in the “Some other heroes of travel” section. Calling him “one of the foremost contributors to our knowledge of South America,” the company noted he had conducted no fewer than seven expeditions across Colombia, Venezuela and Brazil and had “mapped the prodigious area of some 500,000 square miles of tropical South America” (BWC, 1934, p.35). Quite obviously, BWC took the opportunity to provide a summary of Rice’s review of the pharmaceuticals and medical accessories he used in his fifth expedition as follows: “Everything supplied by Burroughs Wellcome & Co. has proved most satisfactory and efficient under most trying and adverse conditions of climate and stubborn cases of disease” (Monthly…, 1919-1921). The company’s records also state that Rice carried another Tabloid kit on his fifth expedition, a Livingstone Medicine Case, most likely for his or his wife’s personal use (Explorers…, 1919).

Both Rice and Wellcome believed that applying science to the quest for natural medicines, production of drugs and promotion of health were the cornerstones of advancing capitalist societies in the tropics. This belief is also revealed in Rice’s desire to leave palpable contributions to improving the health of Amazonian society – all grounded, of course, in the discourse of philanthropy. In Manaus he familiarised himself with the work of Harold Wolferstan Thomas (1875-1931) and Anton Breinl (1880-1944), physicians leading the Manaos Research Laboratory and Hospital, an enterprise of the Liverpool School of Tropical Medicine with support from the Amazonian government. Rice donated his modern motor launch to Thomas, to be used as an itinerant laboratory of endemic diseases and hospital for people living in hard-to-access areas. Rice and his wife also donated funds and began construction of a school in São Gabriel da Cachoeira, in the middle Negro river, claiming that a plan to civilise the Amazon should start with medical assistance based on science and quality education. Rice received high praise from Brazilian Ambassador Domício da Gama (1862-1925), who was present at a RGS session when one of his reports was shared. Gama highlighted Rice’s ideas for the industrial and scientific modernisation of the Amazon, as well as his belief in the Amazonian people’s potential for social progress under appropriate health and educational conditions (Rice, 1920, p.60; Gama, 1921, p.344).

In sum, the background that connected Rice’s exploratory trajectory with the Tabloid medicine chests reveals interesting points related to the interplay between scientific authority, industry, commerce and the cultures of exploration in the first decades of the twentieth century. As Felix Driver (2001, p.8-9) emphasised with regard to the idea of “cultures of exploration,” in nineteenth- and twentieth-century geography (and other fields), exploring meant “a set of cultural practices which involve[d] the mobilization of people and resources, especially equipment, publicity and authority.” Therefore, Rice’s blend of both old-style exploration and new technological geographical surveying methods drew largely from the fact that “the explorer’s search for a reputation depended … upon more than their own labours” (Driver, 2001, p.11). This process also involved being socially well-connected and constant public reference. It is no coincidence that Driver and others referred to the boom in scientific, colonial and touristic expeditions in the late 1800s and early 1900s as “the business of exploration.”

Figures like Hamilton Rice and objects such as medicine chests exemplify the process of transforming scientific and medical exploration into a business. Rice’s approach to scientific exploration and public relations highlights the commercial and cultural dimensions that BWC communicated through the company’s product line of medicine chests, which enabled and sustained these ventures. During Rice’s time, exploration became intertwined with the simultaneous production of knowledge, imperial expansion, and public consumption, mediated through outlets such as maps, reports, popular narratives, exhibitions and advertisements.

## Final considerations

Interesting parallels and connections can be found between the promoted mission of the Tabloid medicine chests and the ideals related to scientific and capitalist modernisation of the Amazon promoted by Hamilton Rice. The background history of Rice’s Tabloid medicine chest exemplifies important dimensions for further discussions about science and capitalism in the early twentieth century. This history includes BWC’s marketing discourse, with the narrative of scientific achievement an intrinsic part of its complex cycle of research, pharmaceutical sales, and collecting their own medicine chests and exhibiting them as historical relics. The BWC’s motto, diversified partnerships around the world, and innovation cemented beliefs in capitalist progress and the British “civilisational mission.”

The details of the Tabloid medicine chest carried by Rice on his fifth Amazonian expedition show that although the case itself and its potential for mobility might be considered pioneering, the medicines cannot. Moreover, these medications demonstrate that the medical imagery of the Amazon region held by both BWC and Rice reflected the oldest notions of “tropicality.” We also saw how Rice’s Amazon Tabloid medicine chest became a science relic, within the context of cultures of exploration, collection and exhibition, with both scientific and economic meaning.

For Rice’s fifth expedition, similarities can be seen between the marketing discourse related to the Tabloid chests and Rice’s self-promoted mission of modernising the Amazon. Although he faced some stumbling blocks in scientific recognition for his geographic accomplishments, his medical achievements involving BWC’s products ensured him considerable respect. Rice’s contributions as an explorer spanned geography, exploration and tropical medicine, intertwining scientific and technological knowledge and practices with philanthropic and capitalist agendas.

## NOTES

1 Wellcome also researched and collected many other kinds of objects related to medicine, pharmacology, and health in general, from different cultures and eras.

2 Indeed, in the marketing industry the set of qualities that make a product interesting, attractive, and reliable is known as “product personality;” other similar terms are “corporate personality” and “brand personality.”

3 A natural thermoplastic that preceded modern industrial plastic and was widely used from the nineteenth century onward for various purposes.

4 Dimensions are 230mm x 400mm x 270mm.

5 Today, the Huitoto or Witoto Indigenous people live between the Amazonas, Caquetá, and Putumayo departments of Colombia, between the Putumayo river and Caquetá, north of Porto Arturo.

6 In Rice’s time, the Yamu people were reportedly living on the left bank of the lower Ariari river in Colombia.
